# Identification of a non-coding RNA and its putative involvement in the regulation of tetanus toxin synthesis in *Clostridium tetani*

**DOI:** 10.1038/s41598-021-83623-0

**Published:** 2021-02-18

**Authors:** Holger Brüggemann, Diana Chapeton-Montes, Lucile Plourde, Michel R. Popoff

**Affiliations:** 1 Department of Biomedicine, Aarhus University, Aarhus, Denmark; 2 Bacterial Toxins, Institut Pasteur, 25 rue du Dr Roux, 75724 Paris Cedex15, France; 3 Sanofi-Pasteur, Marcy l’Etoile, France

**Keywords:** Bacterial toxins, Small RNAs

## Abstract

*Clostridium tetani* produces the tetanus toxin (TeNT), one of the most powerful bacterial toxins known to humankind and responsible for tetanus. The regulation of toxin expression is complex and involves the alternative sigma factor TetR as well as other regulators. Here, a transcriptional analysis of the TeNT-encoding large plasmid of *C. tetani* identified a putative non-coding small RNA (sRNA), located in close vicinity of the 3′ untranslated region of the *tent* gene. A northern blot experiment could identify a respective sRNA with a size of approx. 140 nucleotides. Sequence analysis showed that the sRNA contains a 14-nucleotide region that is complementary to a 5′ located region of *tent.* In order to investigate the function of the sRNA, we applied a RNA interference approach targeting the sRNA in two *C. tetani* wild-type strains; the constructed antisense *C. tetani* strains showed an approx. threefold increase in both extracellular and total TeNT production compared to the respective wild-type strains. In addition, recombinant *C. tetani* strains were constructed that contained *tent-*locus harboring plasmids with and without the sRNA. However, the introduction of the *tent-*locus without the sRNA in a *C. tetani* strain lacking the wild-type TeNT-encoding large plasmid resulted in a lower TeNT production compared to the same strain with recombinant *tent*-locus with the sRNA. This suggests that the expression or the effect of the sRNA is modulated by the *C. tetani* genetic background, notably that of the wild-type TeNT-encoding large plasmid. In addition, some recombinant strains exhibited modulated growth patterns, characterized by premature bacterial cell lysis. Taken together, our data indicate that the sRNA acts as a negative regulator of TeNT synthesis, with a possible impact on the growth of *C. tetani.* We hypothesize that the role of this sRNA is to limit toxin levels in the exponential growth phase in order to prevent premature bacterial lysis.

## Introduction

*Clostridium tetani* is a Gram-positive anaerobic spore-forming bacterium that is widely distributed in the environment. *Clostridium tetani* produces the tetanus toxin (TeNT) that is responsible for tetanus. The mode of action of TeNT has been investigated since several decades. TeNT produced in a necrotic wound contaminated with *C. tetani,* binds to peripheral nerve termini by interacting with specific receptors including polysialogangliosides and nidogens, then undergoes a retrograde transport in motorneurons and enters inhibitory interneurons in the central nervous system, where it cleaves the SNARE protein VAMP resulting in the blockade of neurotransmitter (glycine and GABA) release. The clinical symptoms are characterized by muscular rigidity, painful spasms and autonomic instability^1–4^.

In *C. tetani* strain E88, the *tent* gene is located on a large 74 kb plasmid, pE88^5^. The gene *tetR* is conserved just upstream of *tent*, and encodes for an alternative sigma (σ)-factor, which positively regulates the transcription of *tent*^6^. Furthermore, the 74 kb *C. tetani* plasmid harbors other putative regulatory genes of TeNT synthesis, such as a two-component system (CTP21/CTP22), CTP4 and CTP5 (homologs of *Clostridium perfringens* UviAB that positively control a UV-inducible bacteriocin gene), CTP10 (a putative σ-factor/DNA binding protein), and CTP11 (a putative σ-factor/DNA binding protein)^5^. At least, three two-component systems and the master regulator of metabolism, CodY, control TeNT synthesis^7^. However, the whole regulatory network controlling TeNT production still remains poorly understood.

The importance of regulation by small regulatory RNAs (sRNAs) has been recognized in prokaryotes^8,9^. sRNAs are key players in mediating bacterial responses to environmental signals. In addition, they are important regulators of virulence in several pathogenic bacteria. Furthermore, the regulation by sRNAs is advantageous when fast responses to external signals are needed^10,11^. Sizes of bacterial sRNA are typically between 50 and 500 nucleotides. Regulatory sRNAs have been identified in different genomic localizations. They can be found in or overlapping with 5′ and 3′ untranslated regions (UTRs) next to the coding sequence, in intergenic regions, and as antisense RNAs transcribed on the opposite strand of the open reading frame (ORF)^11,12^. In the majority of cases, antisense RNA results in post-transcriptional inhibition of target mRNA function, but in a few cases, activating mechanisms have been involved^8,13^. Their structures are more stable than those of mRNAs. They begin with a sequence that can fold into a stable stem-loop, and transcription terminates with a rho-independent transcription terminator, a stem-loop that also helps to stabilize the molecule^14,15^.

Regulatory RNAs are divided into (1) *cis*-acting antisense RNAs, which are transcribed from the DNA strand opposite to the target mRNA, and display perfect base complementarities to their targets, and (2) *trans*-acting antisense RNAs, which are transcribed from distant loci from target mRNAs, and are only partially complementary with their mRNA targets. The RNA chaperone Hfq protein (in Gram-negative bacteria) helps target recognition to promote *trans*-antisense RNA binding by an unknown mechanism^11^. Regulatory RNAs are the most abundant class of post-transcriptional regulators. Small non-coding RNAs control their targets through different mechanisms, including: (1) translational inhibition, which can occur by direct blocking of the ribosome-binding site (RBS) or by induction of structural alterations downstream of the RBS; (2) alteration of mRNA stability, whereby both *cis*- and *trans*-acting RNAs can promote RNA degradation; (3) protein sequestration, via regulatory RNA/protein interaction, thus inducing numerous downstream effects; (4) interaction with DNA (CRISPR-RNA, Clustered Regularly Interspaced Short Palindromic Repeats-RNA) and (5) riboswitches, i.e. regulatory RNAs that bind metabolites or environmental cues^8,13^.

Regulatory sRNAs have been identified in clostridia^16–19^. In *C. perfringens*, a small RNA named VR-RNA (VirR regulated RNA) controls the expression of the alpha- (*plc*), kappa- (*colA*) and beta2-toxin genes in a VirR/VirS dependent manner^18,20^. Another regulatory RNA, VirX, controls the levels of theta-toxin (*pfoA)*, *plc*, and *colA* mRNAs independently of the VirR/VirS regulatory cascade^21^.

The goal of this study was to identify and investigate putative regulatory RNAs in the vicinity of *tent* in *C. tetani* and decipher their role in tetanus toxin synthesis. Our results support the identification of a novel sRNA overlapping with the 3′UTR of *tent*, that putatively functions as a negative regulator of TeNT synthesis.

## Results

### Transcriptional profile of the *tent*-containing large plasmid

**Figure 1:**
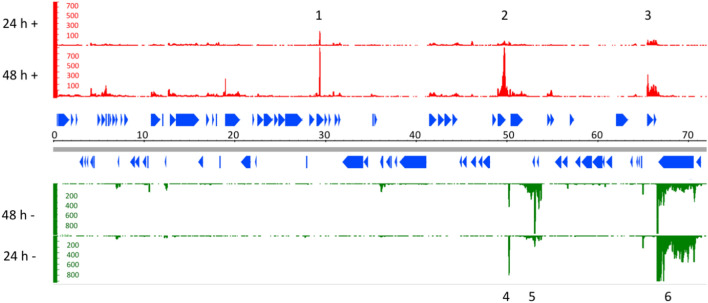
Transcriptome of the *tent*-containing 74 kb plasmid in *C. tetani* strain A when grown under anaerobic conditions in TGY medium for 24 and 48 h. RNA-seq data was mapped to the plus and minus strand of the 74 kb reference plasmid; the nucleotide position is given in kb (grey bar). The blue triangles indicate coding sequences. Peaks of high transcription are labelled: 1, CTP27 (encoding a putative 23S rRNA (guanine-N1) methyltransferase); 2, CTP44 (gene of unknown function); 3, CTP58/CTP59 (genes of unknown function); 4, intergenic region between CTP44 and CTP45 (the latter encodes a putative replication protein); 5, CTP46 and CTP47 (genes of unknown function); 6, *tent* gene (CTP60). The image was created with the Integrative Genome Browser (IGB v8.5.4; https://bioviz.org/).

We first recorded the transcriptome of the *tent*-containing 74 kb plasmid in *C. tetani* strain A when grown under anaerobic conditions in TGY medium for 24 and 48 h (Fig. 1). A few plasmid regions were highly transcribed. On the plus strand, there were strong transcription peaks within genes CTP27 encoding a putative 23S rRNA (guanine-N1-methyltransferase), CTP44 (protein of unknown function) and CTP58/CTP59 (proteins of unknown function). On the minus strand, strongest transcription was associated with an intergenic region between CTP44 and CTP45, the latter encodes a putative replication protein, and with the genes CTP46 and CTP47 (proteins of unknown function). By far the strongest transcription was associated with *tent* (CTP60), in particular at 24 h of growth. Regarding other putative regulatory genes located on the plasmid, we noticed very weak transcription of the two genes that encode putative RNA polymerase sigma factors (CTP10, CTP11), and also weak expression of the putative two-component system (CTP21/CTP22).

### A 3′UTR region of *tent* is transcribed in *C. tetani*

**Figure 2:**
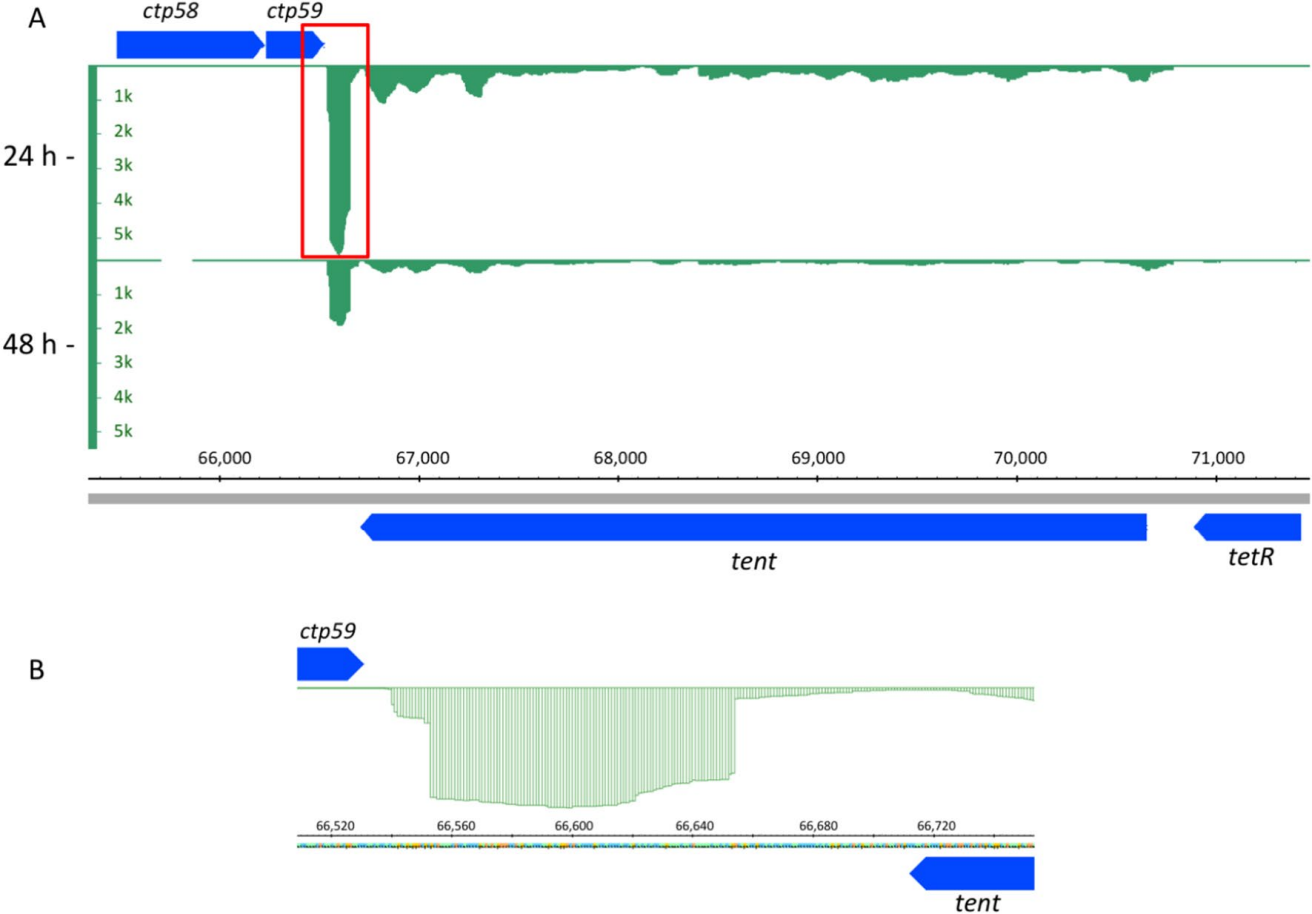
Transcriptional profile of a sRNA downstream of *tent.* (**A**) A plasmid region located downstream of the protein-coding part of *tent* was highly transcribed in *C. tetani* grown for 24 h in TGY medium. (**B**) An enlarged cutout from 2A (red box) illustrates the exact region of the putative sRNA. CDS are indicated in blue. The nucleotide position of the 74 kb plasmid is given in bp (grey bar). The image was created with the Integrative Genome Browser (IGB v8.5.4; https://bioviz.org/).

A closer inspection of the transcriptional profile of *tent* revealed a strong transcriptional peak on the minus strand, located downstream of the protein-encoding part of *tent* and downstream of the *ctp59* gene located on the plus strand (Fig. 2). The transcriptional peak of 114 nt (exact coordinates 66,540–66,654 nt of the plasmid) seems to be located in, or overlapping with the 3′UTR region of *tent*, since transcription continued downstream of the protein-encoding part of *tent*. In order to exclude the possibility that this peak represents a mRNA from a small peptide-encoding gene, we performed a bioinformatics analysis of the six ORFs in the area between *tent* and *ctp59*, but could not detect any likely candidates for peptide-encoding CDS overlapping with the region of the transcriptional peak. We then hypothesized that this peak corresponds to a putative non-coding sRNA. Its location in close vicinity of *tent* could indicate that it is involved in controlling TeNT synthesis.

The 114 nt sequence of the putative sRNA was identified also in all other plasmid-positive Harvard-derived strains clade 1A strains, according to^22^, such as E88, CN655, and strain A with a 100% identity on nucleotide level. In addition, all plasmid-positive *C. tetani* strains of clades 1B to 1E carry an identical sequence (with strain TMB2, a clade 1B strain, as the only exception) (Sup. Fig. S1). In the other *C. tetani* strains, i.e. strains of clades 1F to 1H and clade 2, the sequence is present with four SNPs. We used the Rfam database (a collection of sRNA families) to search whether the putative sRNA has homology to known sRNAs^23^, but no hit was found.

**Figure 3:**
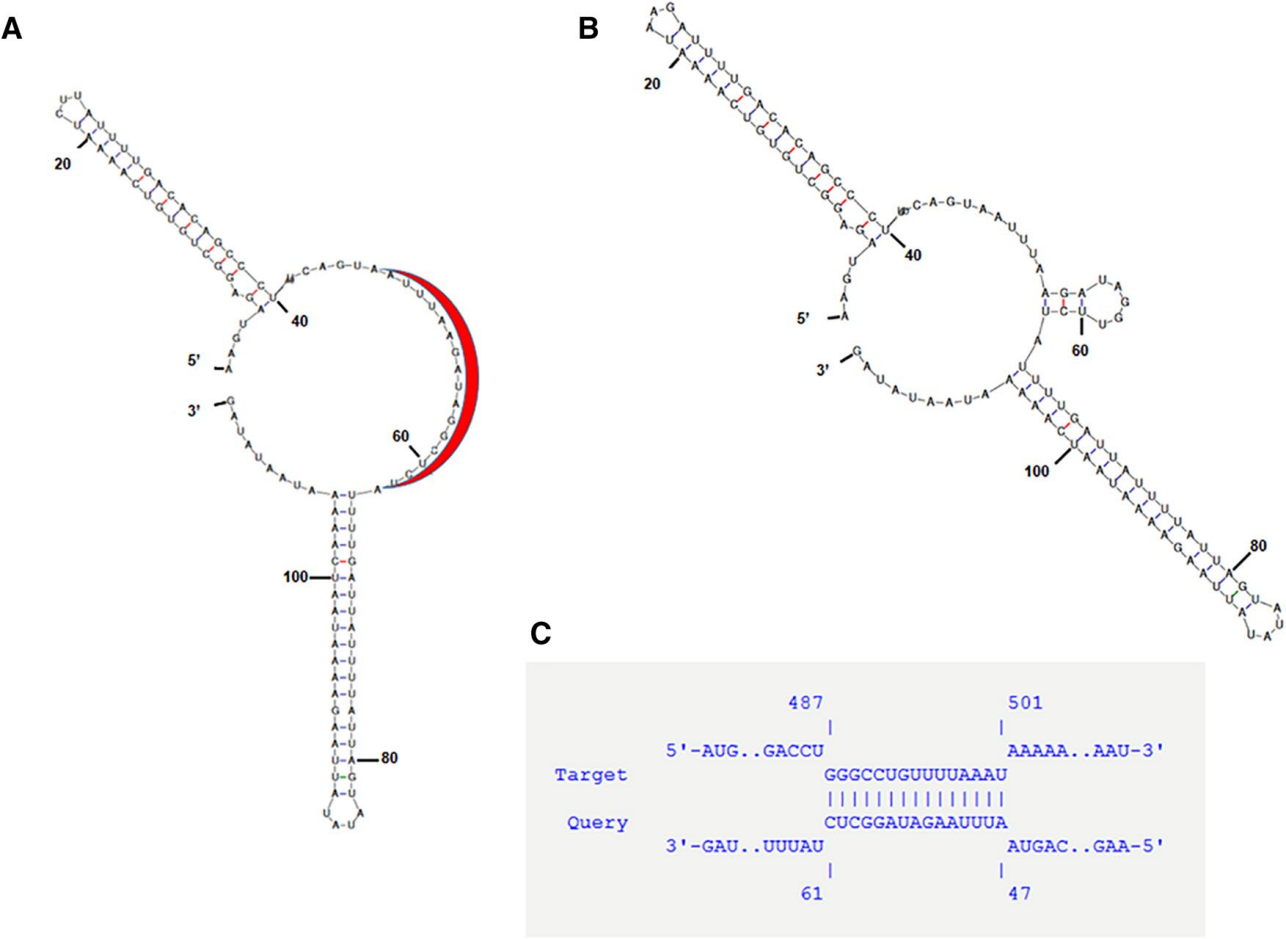
Predicted secondary structure of the putative sRNA of *C. tetani* and its potential binding site. (**A**) Mfold prediction of the sRNA in most *C. tetani* clade 1 strains, including the Harvard derived strains. The red arc represents the potential binding site with *tent.* The stem-loop that is most distant from *tent* contains a Rho-independent terminator, according to an ARNold analysis. (**B**) Mfold prediction of the sRNA from *C. tetani* clade 2 and 1G strains. (**C**) A nearly perfect 14 nt match of the interior junction-loop-exposed sequence (nucleotide positions 47–61) of the sRNA with the 5′ end of *tent* mRNA (nucleotide positions 487–501 of *tent*) was detected. The image was created with mfold (v3.5; http://www.unafold.org/).

According to RNA folding predictions, the putative sRNA of clade 1 strains forms two stem-loop structures with one big interior junction-loop (Fig. 3A). In clade 2 and clade 1G strains, the RNA folds differently, with an additional loop within the interior junction-loop (Fig. 3B). The stem-loop that is most distant from *tent* contains a Rho-independent terminator as judged from an ARNold analysis^24,25^. This is in agreement with other sRNAs, as transcription of most sRNA genes is terminated by Rho-independent termination^14^.

We hypothesize that the secondary structure could potentially sequester *tent*. The IntaRNA tool was used to search for a potential target of this sRNA. The most significant match was found between the interior junction-loop-exposed sequence (between position 47 and 61) of the putative sRNA and a region in the 5′end coding sequence of *tent* (Fig. 3C). This could indicate a direct interaction of the putative sRNA with the *tent* mRNA.

### Expression of the putative sRNA downstream of *tent*

**Figure 4:**
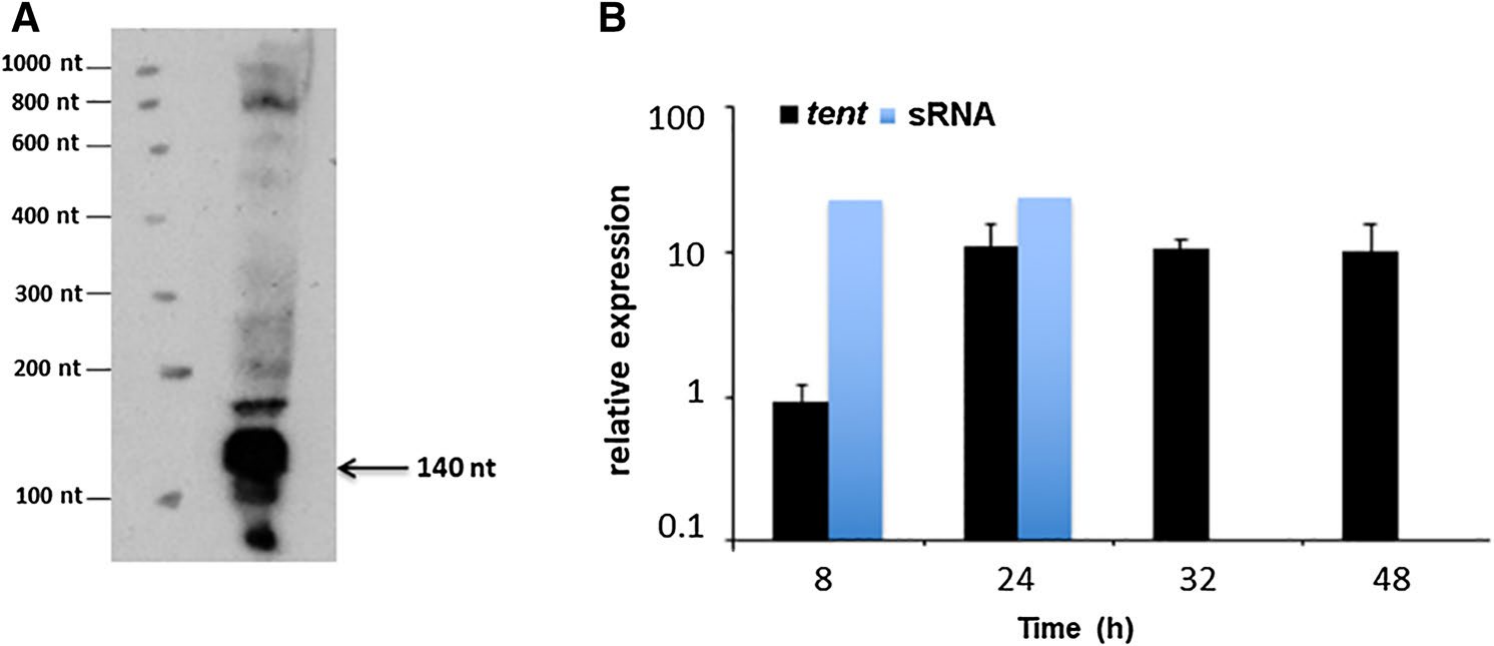
Detection of sRNA by Northern blot in *C. tetani* and its relative expression. (**A**) Northern blot analysis. The arrow points to the detected transcript with its size estimated by comparison with RNA molecular weight standards. Total RNA was extracted from *C. tetani* CN655 grown 24 h in TGY. sRNA was detected in Northern blot with biotinylated P2275 (TAATCAAAATAGAGCCTATC). (**B**) Relative expression in logarithmic scale of *tent* and sRNA in *C. tetani* strain CN655 grown in TGY for the indicated times.

The existence and expression of the putative sRNA was investigated using Northern blot analysis and qRT-PCR, respectively. Northern blot analysis could identify the sRNA as an abundant RNA molecule of approx.140 nt, which is in accordance with the predicted size deduced from RNA-seq data (Fig. 4A). An additional less abundant longer transcript (approx. 170 nt) was also detected and, albeit to a much lower extent, other longer transcripts in a scaled partition that could correspond to multimers. Expression of the sRNA by qRT-PCR was determined in *C. tetani* CN655 grown in TGY media for 24 h and 48 h. This analysis revealed that the sRNA was abundantly expressed, but only at early time points (Fig. 4B). RNA-seq confirmed a weaker transcription of the sRNA at 48 h compared to 24 h of culture (Fig. 2).

### The sRNA modulates tetanus toxin production in *C. tetani*

**Figure 5:**
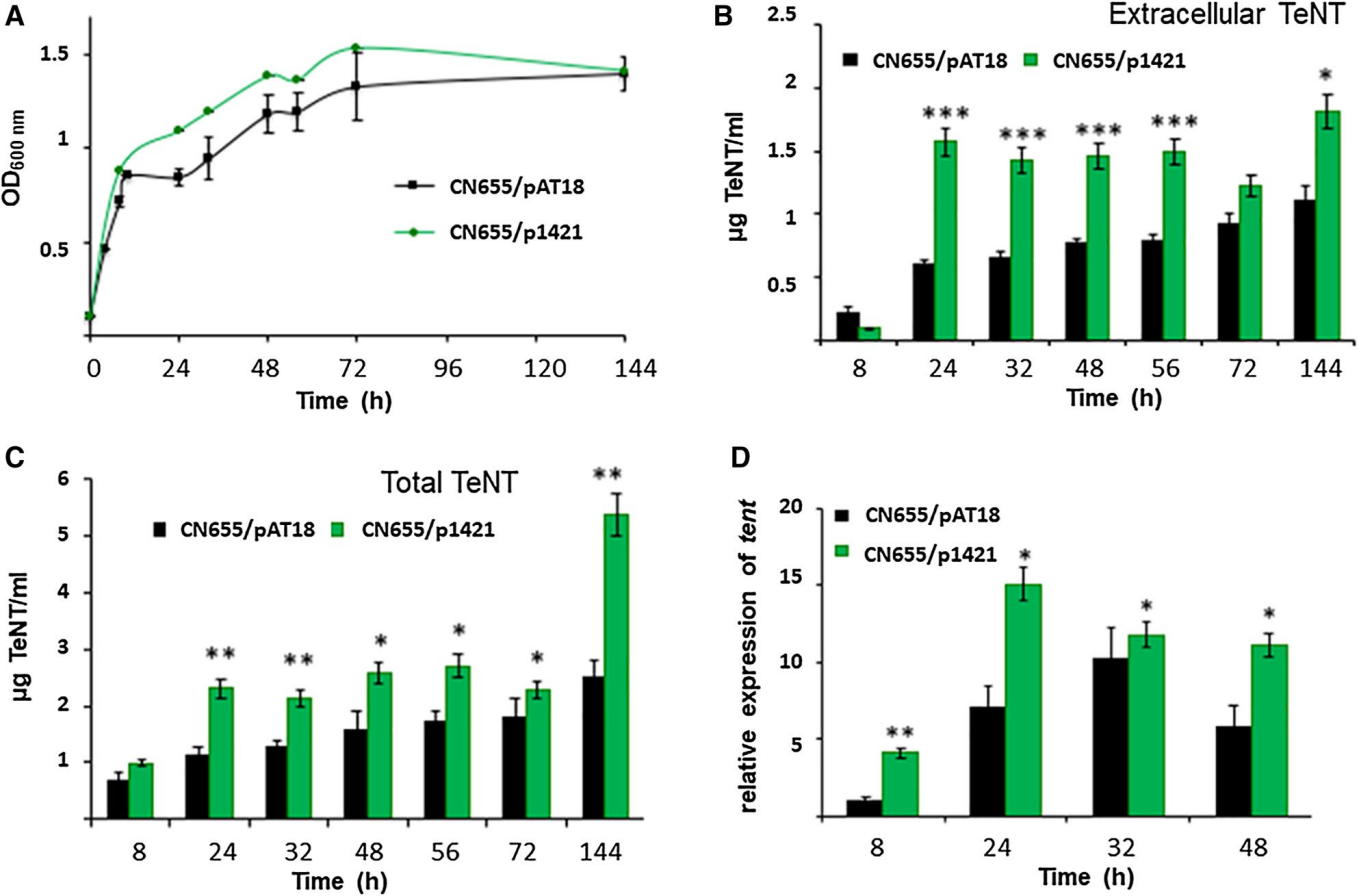
Effect of introducing an antisense sRNA construct (p1421) on growth, TeNT production, and *tent* expression in *C. tetani* CN655. (**A**) Growth kinetics in TGY medium of the recombinant CN655/p1421 (antisense sRNA construct) strain was similar to that of the control strain CN655/pAT18 (control vector). (**B**) Extracellular TeNT was increased in the culture supernatant of CN655/p1421 (antisense sRNA construct) compared to CN655/pAT18 (control vector). (**C**) Total TeNT production was increased in CN655/p1421 (antisense sRNA construct) compared to CN655/pAT18 (control vector). (**D**) Expression of *tent* was increased in CN655/p1421 (antisense sRNA construct) compared to CN655/pAT18 (control vector). Target gene expression was normalized to *rpoB* and *gyrA.* Statistical significance of differences between the control strain and recombinant strains is indicated with p-values (*P < 0.05; **P < 0.01; ***P < 0.001). The data are from three independent experiments.

In order to investigate the regulatory function of the sRNA on toxin synthesis, we constructed a sRNA antisense strain, using an RNA antisense system that has previously been employed in *Clostridium botulinum* and *C. tetani*^6,26^. The plasmid construct with the antisense sRNA was named p1421 (Sup. Fig. S2). The construct was transformed into the wild-type strain CN655, a TeNT-producing clade 1A strain. The recombinant strain targeting the sRNA (CN655/p1421) showed a strong significant increase in both extracellular and total TeNT production at most time points of the exponential and stationary growth phases (Fig. 5). At 24 h, extracellular TeNT levels were threefold increased in the CN655/p1421 strain compared to the control strain CN655/pAT18 (Fig. 5B). In addition, fivefold increased *tent* transcription in the early growth phase (8 h) was observed in strain CN655/p1421compared to the control strain CN655/pAT18 (Fig. 5D). It is noteworthy that the changes in TeNT synthesis were of a larger extend than those in *tent* mRNA levels, suggesting additional post-transcriptional regulation. Taken together, this indicates that the sRNA acts as a negative regulator in *C. tetani* in the early growth phase, leading to decreased TeNT production.

**Figure 6:**
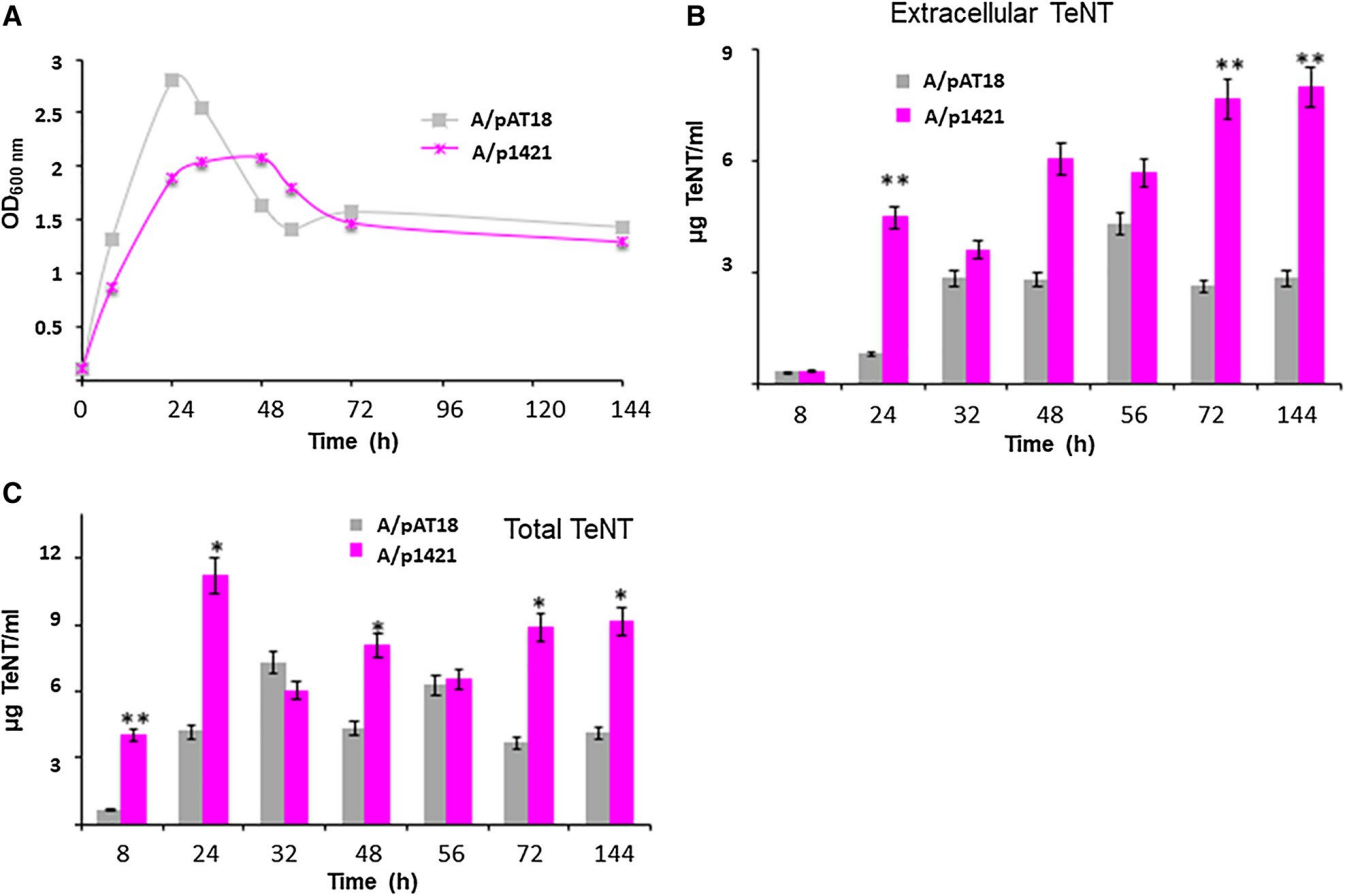
Effect of introducing an antisense sRNA construct (p1421) on growth, TeNT production, and *tent* expression in *C. tetani* strain A. (**A**) Growth kinetics of the recombinant strain A/p1421 (antisense sRNA construct) showed a slower growth and a reduced bacterial lysis after 24 h of culture in TGY medium compared to the control strain A/pAT18. (**B**) Extracellular TeNT was significantly increased in strain A/p1421 compared to the control strain A/pAT18. (**C**) Total TeNT production was increased in strain A/p1421 compared to the control strain A/pAT18. Statistical significance of differences between the control strain and recombinant strains is indicated with p-values (*P < 0.05; **P < 0.01). The data are from three independent experiments.

The RNA antisense system targeting the sRNA was also tested in another Harvard derivative clade 1A strain, *C. tetani* strain A (Fig. 6). This strain produces high amounts of TeNT (three- to fivefold more TeNT production than CN655 in TGY culture, data not shown) and its growth profile is characterized by a rapid growth until approx. 24 h, followed by substantial bacterial lysis from 24 to 56 h and then by a stationary phase (56–144 h) (Fig. 6A). Similar to strain CN655/p1421 (Fig. 5), extracellular TeNT levels and total TeNT production were increased in strain A/p1421 cultures compared to the control strain A/pAT18 (Fig. 6B,C). However in the strain A, the exponential growth and bacterial lysis after 48 h of culture were less pronounced in the recombinant strain A/p1421 than in the control A/pAT18 (Fig. 6A).

### Expression of recombinant *tent* loci with and without sRNA in *C. tetani*

To further investigate the influence of the sRNA in *C. tetani,* the region corresponding to *tetR-tent* with their own promoters and the sRNA, and *tetR-tent* with their own promoters but lacking the sRNA were cloned into the shuttle vector pAT18, yielding p1423 and p1424, respectively (Sup. Fig. S2). We took advantage of the strain 1586-Z1, which is a non-toxigenic derivative of the clade 1A strain 1586-U1^22^. Both strains, 1586-Z1 and 1586-U1, retain the same chromosomal genome sequence, but 1586-Z1 lacks the wild-type large plasmid harboring *tent*^22^. The recombinant plasmids p1423 and p1424 were transformed into 1586-Z1 to compare the expression of the recombinant *tetR-tent* genes, with and without the putative sRNA in a *C. tetani* genomic background.

**Figure 7:**
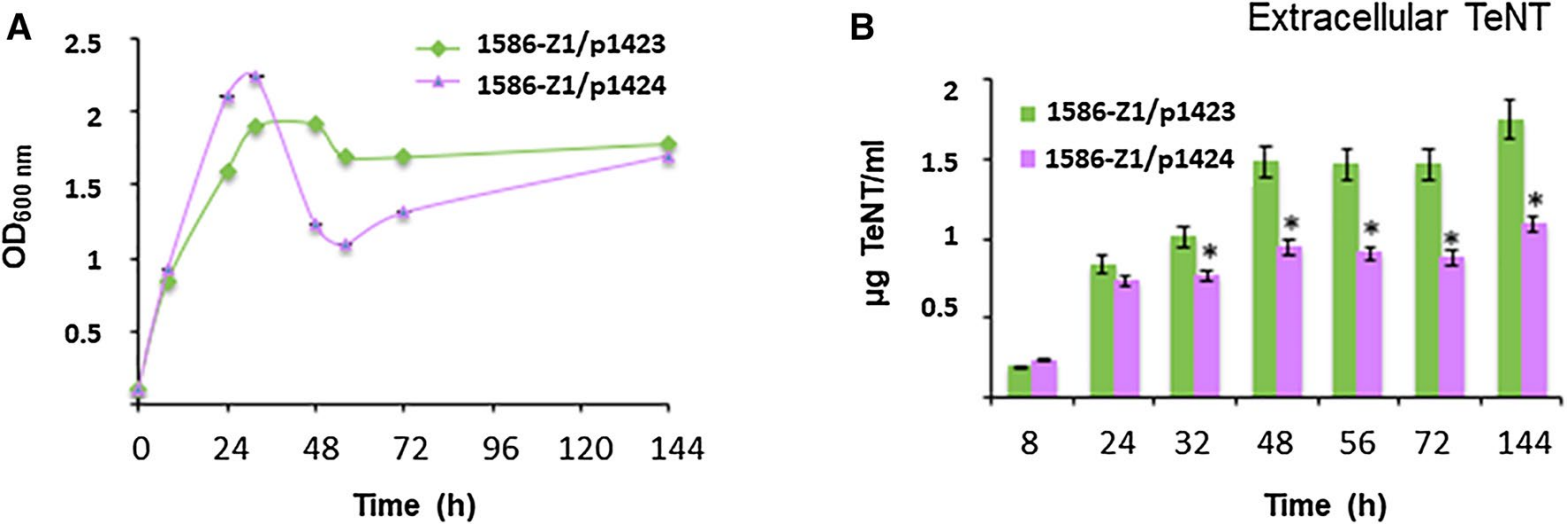
Growth and TeNT production in recombinant *C. tetani* strain 1586-Z1 (lacking the wild-type large *tent* plasmid), harboring the plasmid constructs p1423 (*tent* locus with sRNA) and p1424 (*tent* locus without sRNA). (**A**) Growth kinetics of *C. tetani* 1586-Z1/p1423 (containing *tetR* and *tent* genes with sRNA) and 1586-Z1/p1424 (containing *tetR* and *tent* genes without sRNA) in TGY medium. (**B**) Extracellular TeNT levels were higher in strain 1586-Z1/p1423 than in strain 1586-Z1/p1424. Statistical significance of differences between the two recombinant strains is indicated with p-values (*P < 0.05; **P < 0.01). The data are from three independent experiments.

TeNT production was observed in 1586-Z1 transformed with p1423 or p1424. High levels of extracellular TeNT were measured in 1586-Z1 when p1423 containing functional copies of *tetR, tent* and sRNA (Fig. 7B). Extracellular TeNT levels in 1586-Z1/p1423 were in a similar range than in CN655/p1421 (Fig. 5B). Unexpectedly, 1586-Z1/p1424 (containing functional copies of *tetR, tent* but lacking the sRNA) showed lower levels of extracellular TeNT in the late growth phase (32–144 h) compared to strain 1586-Z1/p1423 (Fig. 7B). However, the strain 1586-U1 (which contains the wild-type TeNT-encoding large plasmid), transformed with p1423 and p1424 showed a different result. In contrast to 1586-Z1, the extracellular TeNT production was higher in 1586-U1/p1424 (containing functional copies of *tetR, tent* but lacking the sRNA) than in 1586-U1/p1423 (containing functional copies of *tetR, tent* and sRNA) (Sup. Fig. S3), thus exhibiting a similar pattern than strain A/p1421 (Fig. 6B). The apparently discrepant observation might result from different copy number ratios of *tetR-tent* genes and sRNA in strains with and without the wild-type TeNT-encoding large plasmid.

## Discussion

Here, we show that the large plasmid in toxigenic *C. tetani* strains contains a conserved region downstream of the protein-coding sequence of *tent.* This region within or overlapping with the 3′UTR of *tent* encodes a sRNA of approx. 140 nucleotides that was highly expressed in the exponential growth phase. We found that this sRNA negatively regulated the expression of *tent* and TeNT synthesis, as judged from experiments using a RNA interference system targeting the sRNA in toxigenic clade 1A *C. tetani* strains. The inhibitory regulatory role of the sRNA on *tent* expression is likely due to sequestration of *tent* mRNA, since a complementary sequence of the interior junction-loop-exposed sequence of the sRNA was found in the 5′ end of the protein-coding part of *tent* (Fig. 3). It is noteworthy that the sRNA exhibited an imperfect secondary structure in clade 2 *C. tetani* strains (Fig. 3B) that might result in a weaker effect. The inhibitory regulatory role of the sRNA in clade 1A *C. tetani* strains is further supported by experiments showing increased levels of *tent* transcripts in the early exponential growth of recombinant strains containing the antisense sRNA construct as well as higher extracellular TeNT production (Figs. 5 and 6). The lower TeNT synthesis in 1586-Z1/p1424 (recombinant *tetR-tent* lacking the sRNA) versus in strain 1586-Z1/p1423 (*tetR-tent-*sRNA) is probably due to the absence of the 3′UTR of *tent* (51 nucleotides between the *tent* stop codon and sRNA) in the p1424 construction. Indeed, 3′UTRs of genes are known to contain terminator and regulatory sequences that can influence gene expression and RNA stability^27,28^. Alternatively, the apparently different results in strains lacking the sRNA (i.e. 1586-Z1/p1424 compared to the antisense strain containing p1421) might be related to the high copy number of the recombinant plasmids versus the low copy number of the wild-type large plasmid harboring *tent*. *tent* mRNA levels have not been quantified in strains 1586-Z1 and 1586-U1 transformed with p1423 or p1424.

Besides the profound impact on TeNT synthesis, the sRNA downstream of *tent* may be involved in the modulation of *C. tetani* growth. Altering sRNA levels in cells often lead to aberrant growth kinetics^29,30^. A first indication came from the antisense experiments in strain A (Fig. 6). Introducing the antisense plasmid p1421 significantly altered the growth kinetics. Further evidence came from additional experiments, e.g. the transformation of the *tent* locus lacking the sRNA (p1424) into the *C. tetani* strain 1586-Z1. This resulted in a growth pattern that is characterized by an abundant exponential growth phase followed by substantial bacterial lysis in the early stationary phase, while *C. tetani* strain 1586-Z1 harbouring p1423 showed a growth pattern similar to that of the wild-type strain CN655 (Fig. 7). In addition, introducing p1424 into strain CN655 resulted also in a growth pattern that was characterized by premature bacterial lysis (Sup. Fig. S4). In contrast, the transformation of p1423 (*tetR-tent-*sRNA) in CN655 did not modify the growth pattern of CN655 and only induced a transient over-expression of *tent* in the early growth phase without significant increase of extracellular TeNT (Sup. Fig. S4). These results are difficult to interpret for each individual strain, due to the simultaneous expression of recombinant *tent* loci with and without the sRNA (located on the plasmids p1423 and p1424) and the wild-type *tent* locus (located on the large plasmid). However, taken together these results indicate that the sRNA seems to contribute to the control of the growth pattern of *C. tetani*, either directly or indirectly. Indirect regulatory effects could be mediated through genes from the metabolic pathway or bacterial cell wall synthesis. The absence of sRNA in the *tent* locus seems to facilitate a growth pattern with an abundant exponential growth phase and premature bacterial lysis in the early stationary phase. Thus, this sRNA might have pleiotropic effects in analogy to other sRNAs. For instance, in *C. perfringens,* the regulatory RNA (VR-RNA) is involved in a regulatory cascade controlling 147 genes including toxin and virulence genes such as alpha-toxin, kappa-toxin, hyaluronidases, sialidase, and capsular polysaccharide synthesis^31,32^.

In conclusion, a sRNA lies downstream of *tent* and is expressed concomitantly with *tent.* The sRNA downregulated the expression of *tent,* likely by antisense sequestration of *tent* mRNA, and modulated the growth pattern of *C. tetani*. The interlocked activity of the sRNA regarding TeNT synthesis and bacterial growth might prevent excessive TeNT production during the exponential growth phase, thus facilitating the expansion of *C. tetani*. Unraveling the regulatory network of TeNT synthesis would allow a better understanding of the pathogenesis of tetanus as well as the in situ and in vitro production of TeNT.

## Materials and methods

### Plasmid and bacterial strain construction and culture conditions

*C. tetani* strains used in this study were: A, CN655, and 1586-Z1, a derivate of 1586-U1, but without the large *tent*-harboring plasmid^22^. Recombinant *Escherichia coli* BL21 strains were grown in Luria–Bertani (LB) broth, and *C. tetani* strains in TGY (trypticase, yeast extract, glucose) broth (pH 7.5), under anaerobic conditions^7^. When necessary, erythromycin was added to culture media at 5 or 50 µg/ml for *C. tetani* mutants and 300 µg/ml for *E. coli*.

The pAT18 vector was used for genetic manipulation of *C. tetani*^6^. To investigate the effect of the putative sRNA, the sRNA-encoding DNA fragment was amplified by PCR with P2300 and P230 adding a *Pst*I and *Nco*I site, respectively. The PCR-amplified sRNA was inserted in reverse orientation into pMRP306^26^. The respective plasmid was named p1421 (Sup. Fig. S2). In addition, two recombinant plasmids were built: p1423 (pAT18/*tetR-tent* including the sRNA) and p1424 (pAT18/*tetR-tent* without the sRNA) (Sup. Fig. S2). The primers used to PCR amplify *tetR-tent-*sRNA and *tetR-tent* are listed in Sup. Table S1. The recombinant plasmids p1421, p1423 and p1424 were transformed by electroporation into strains CN655, A, 1586-U1 and 1586-Z1. The transformants were selected on erythromycin (5 μg/ml) TGY agar plates.

### Tetanus toxin assay

At 8, 24, 32, 48, 56, 72 and 144 h of growth, 4 ml of culture were removed. The cells were harvested at 22,000 RCF for 10 min at 4 °C, and the supernatants corresponding to the extracellular toxin were filtered (0.22 μm) and stored at − 20 °C. In order to recover intracellular toxin, the pellets were washed with water and osmotic lysis was performed by homogenization and incubation in TGY containing 20 mg/ml NaCl and 13.3 mg/ml sodium citrate (C_6_H_5_Na_3_O_7_, 2H_2_O) during 24 h at 4 °C. After centrifugation, the pellet was lysed again in the same procedure and same conditions. Finally, supernatants were filtered and stored at − 20 °C.

Extracellular and intracellular TeNT levels were determined by an enzyme-linked immunosorbent assay (ELISA) as previously described^7^.

### Total RNA extraction, reverse transcription and quantitative real-time PCR analysis

Total RNA from *C. tetani* strains were extracted at 8, 24, 32, and 48 h of growth, extracted, treated with DNAse (TURBO DNA-free kit, Ambion), and converted in cDNAs as previously described^7^.

Real-time quantitative RT-PCR was performed in duplicate in a 25 µl reaction volume containing 30 ng of cDNAs, 12.5 µl of SYBR Green Supermix (Bio-Rad, 2X; 1,25 U iTaq DNA polymerase, 0.4 mM each dNTP, 6 mM MgCl_2_, 20 nM fluorescein, SYBR Green I) and 500 nM gene-specific primers (Sup. Table S1) in an iQ iCycler apparatus (Bio-Rad). *rpoB* and *gyrA* were used as an internal reference as previously described^7^.

The relative cDNA quantity of each sample was determined with threshold cycle [ΔΔCT] method^33,34^. cDNA quantity of *tent* and *tetR* genes was normalized to the quantity of cDNA of the *rpoB* and *gyrA* gene. Primers used were as follows: *tent*, forward 5′-CCAAGGTGCACAAGGAATTT-3′ and reverse 5′- CAATGTTTAATGCGGGTCCT-3′; *gyrA*, forward 5′- AAGATGATGTAGCAGTAAGTATGGA-3′ and reverse 5′- CTCTGAAGCCAATGTCCTTTT-3′; and *rpoB*, forward 5′-TTGAAGAATGTAAAGAGAGAGATGCTAC-3′ and reverse 5′- GGGAAGTCACCCATAAAGACA-3’.

### Northern blot

Northern blot analysis was performed using North2South Chemiluminescent Hybridization and Detection Kit (Thermo Scientific) with biotin oligonucleotides probes according to manufacturer's recommendations. Briefly, total RNA samples (5 μg) were run in a 10% denaturing polyacrylamide gel with 8 M urea in 1 × TBE (Tris–borate-EDTA buffer). The RNA gels were transferred to Hybond-N + (Amersham Biosciences) at 50 V (60–90 min) using a Trans-Blot transfer cell (Bio-Rad). The RNA was fixed onto the membrane by a UV cross-linking. Prehybridization was carried out for 1 h at 55 °C in North2South Hybridization Buffer. Hybridization was performed overnight at 55 °C in the same buffer with biotin-labeled DNA oligonucleotide probe and membranes were washed. The size of the transcripts was estimated by comparison with RNA molecular weight standards (Invitrogen). Then, the membranes were washed twice for 5 min in 2 × SSC (300 mM sodium chloride and 30 mM sodium citrate) 0.1% sodium dodecyl sulphate (SDS) buffer and twice for 15 min in 0.1 × SSC 0.1% SDS buffer. Labeled probes were detected with streptavidin-HRP and the Chemiluminescent Nucleic Acid Detection Module (Thermo Scientific).

### RNA seq

Total RNA was extracted as described above. The cDNA libraries were constructed by Vertis Biotechnology AG, Germany, as previously described^35,36^. The cDNA libraries were sequenced using a HiSeq 2000 instrument (Illumina) in a single-read mode and 100 cycles. Detailed description of procedures used for quality control, read mapping, expression graph construction and normalization of expression graphs have been published previously^35^. For graph visualization the Integrative Genome Browser (IGB v8.5.4) was used^37^.

DNA sequences are from genomes of *C. tetani* strain A (GenBank JWIX00000000) and *C. tetani* CN655 (GenBank (JSWC00000000). The RNA sequences discussed in this publication have been deposited in NCBI's Gene Express Omnibus^38^ and are accessible through GEO series accession number GSE150141.

### Statistics

Values throughout the manuscript are expressed as means ± standard error of the mean. Differences in the different isogenic anti-sense strains were assessed using unpaired Student’s t-test where statistical significance is assumed for *P < 0.05, **P < 0.01, and ***P < 0.001.

## Supplementary Information

Supplementary Information
