# Supplementary material for: Identification of a non-coding RNA and its putative involvement in the regulation of tetanus toxin synthesis in Clostridium tetani
Source: Sci Rep. 2021 Feb 18;11:4157. doi: 10.1038/s41598-021-83623-0 (PMC7892561; doi:10.1038/s41598-021-83623-0)
Supplement: Supplementary file 1 — Supplementary Information [file 41598_2021_83623_MOESM1_ESM.docx]

**Identification of a non-coding RNA and its putative involvement in the regulation of tetanus toxin synthesis in *Clostridium tetani***

**Holger Brüggemann^1a^, Diana Chapeton-Montes^2a^, Lucile Plourde^3^, Michel R. Popoff^2*^**

^1^Aarhus University, Department of Biomedicine, Aarhus, Denmark

^2^Bacterial Toxins, Institut Pasteur, Paris, France

^3^ Sanofi-Pasteur, Marcy l'Etoile, France

**Supplementary information**

**Supplementary Figure 1. Phylogenetic tree of the predicted sRNA in *C. tetani* strains.** The 114-nt sRNA was found in all plasmid-positive *C. tetani* strains. There are two main variants; one variant is shared by all Harvard-derived strains (clade 1A) and strains of clades 1B to 1E. A second variant with four SNPs is present in strains of clades 1F to 1H and clade 2.

**Supplementary Figure 2. Schematic representation of the recombinant plasmids.** (A) Plasmid p1421 contains the DNA encoding the sRNA antisense cloned into pMRP306, a derivative of plasmid pAT18, that contains the constitutive promoter (*P_iota_*) and 3'-part of the iota toxin gene ^1,2^. pAT18 is a shuttle vector between *Escherichia coli* and Gram-positive bacteria which contains replication origins for *E. coli* and Gram-positive bacteria, a multiple cloning site, the *lacZα* reporter gene, and an erythromycin-resistance gene ^3^. (B) The plasmid p1423 contains the DNA encoding *tetR* and *tent* under the control of their own promoters *P_tetR_* and *P_tent_* respectively, and with the 3' part encoding sRNA. The corresponding DNA fragment was PCR-amplified with primers P1736 and P1737 and cloned into pAT18. (C) The plasmid p1424 contains the DNA encoding *tetR* and *tent* under the control of their own promoters but without the 3'-part encoding sRNA. The corresponding DNA fragment was PCR-amplified with the primers P2300 and P2301 and cloned into pAT18.

**Supplementary Figure 3. Growth pattern and extracellular TeNT production of *C. tetani* 1586-U1 transformed with p1423 or p1424.** (A) Similar growth kinetics were observed in *C. tetani* 1586-U1/p1523 and 1586-U1/p1524. (B) Higher extracellular TeNT levels were monitored in *C. tetani* 1586-U1/p1424 compared to 1586-U1/p1423. Data are from at least three independent experiments.

**Supplementary Figure 4.** **Growth pattern and extracellular TeNT production of *C. tetani* CN655 transformed with p1423 and p1424.** (A) Growth kinetics of CN655/pAT18, CN655/p1423, and CN655/p1424. Extracellular (B) and total TeNT (C) levels in CN655/pAT18, CN655/p1423, and CN655/p1424. (D) Relative *tent* expression in CN655/pAT18, CN655/p1423, and CN655/p1424. Statistical significance of differences between the control strain and mutant is indicated with p-values (*, *P*<0.05; **, *P*<0.01). Data are from at least three independent experiments.

**References**

1 Marvaud, J. C. *et al.* *bot*R is a positive regulator of botulinum neurotoxin and associated non toxic protein genes in *Clostridium botulinum* A. *Mol. Microbiol.* **29**, 1009-1018 (1998).

2 Perelle, S., Gibert, M., Boquet, P. & Popoff, M. R. Characterization of *Clostridium perfringens* iota toxin genes ans expression in *Escherichia coli*. *Infect. Immun.* **61**, 5147-5156 (1993).

3 Trieu-Cuot, P., Carlier, C., Poyart-Salmeron, C. & Courvalin, P. Shuttle vectors containing a multiple cloning site and a *lacZa* gene for conjugal transfer of DNA from *Escherichia coli* to gram-positive bacteria. *Gene* **102**, 99-104 (1991).

**Supplementary Fig. 1**

**
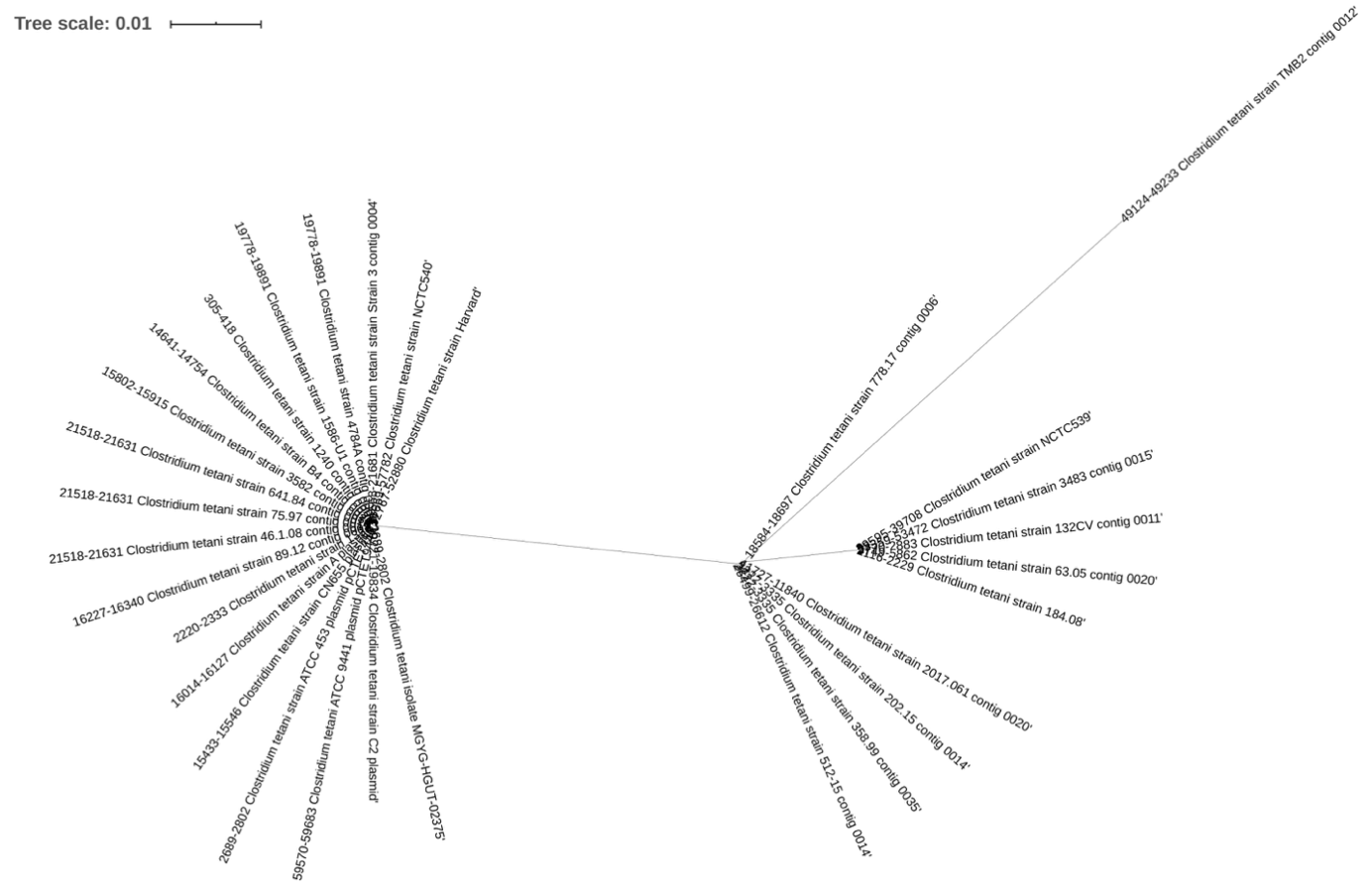
**

**Supplementary Fig. 2**

**
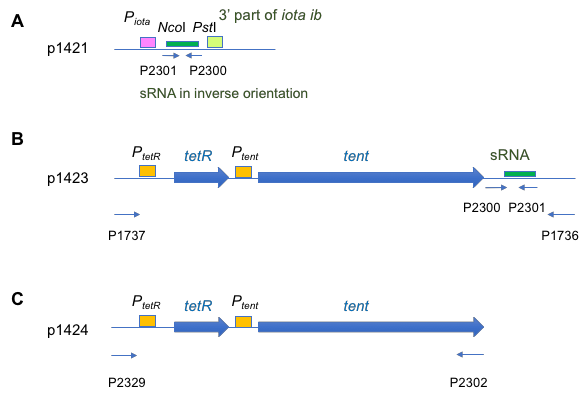
**

**Supplementary Fig. 3**

**
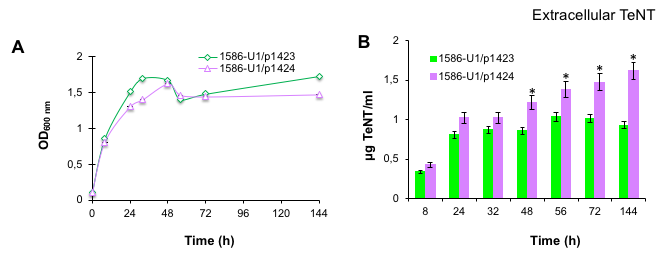
**

**Supplementary Fig. 4**

**
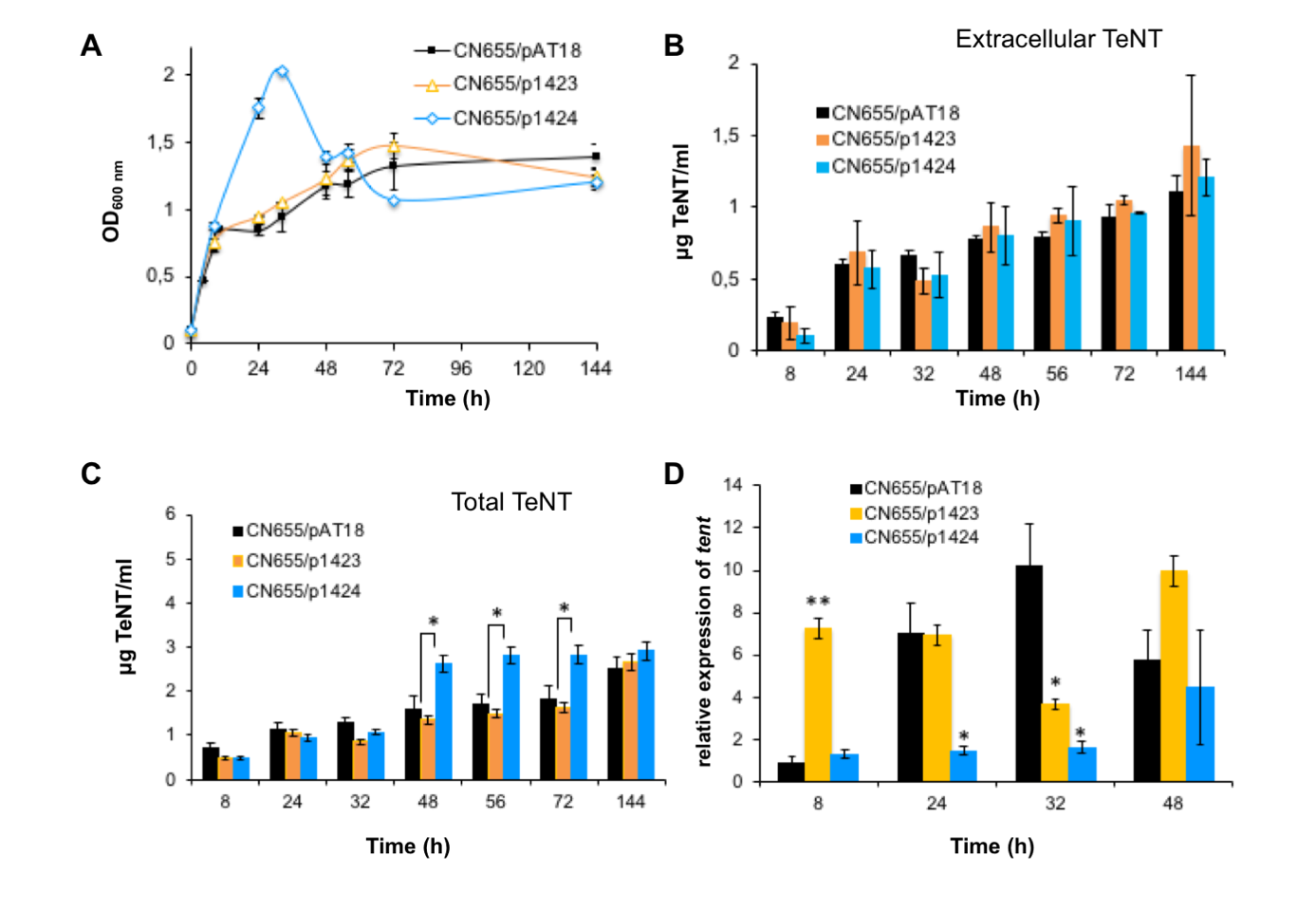
**

**Supplementary Table 1. Primers and recombinant plasmids**

| Plasmid | Primer | Primer sequence | Primer positioning | plasmid characteristics |
| --- | --- | --- | --- | --- |
| p1424 | P2302 | ctgcagTTAATCATTTGTCCATCCTTC | 3' *tent* | *tent* with promoter and without sRNA |
|  | P2329 | gcatgcGAATAATATTCTTAAGATAAAAAG | 5' *tent* promoter |  |
| p1423 | P1736 | ctgcagTACTATATTAAATCTAATACATGC | 3' nc-RNA | *tent* with promoter and sRNA |
|  | P1737 | ggatccGAATAATATTCTTAAGATAAAAAG | 5' *tent* promoter |  |
| p1421 | P2300 | ccgctgcagACAGATTGATATGTTCATGA | 5' nc-RNA | antisense sRNA |
|  | P2301 | ggccatggTATTATAAGTAGAGGCTG | 3' nc-RNA |  |
|  | | | | |
| Primers for qPCR | | | | |
|  | P2275 | TAATCAAAATAGAGCCTATC | detection of sRNA | |
|  | P2276 | AATCTTATTTTGACACAGCC |  |  |
|  | P1714 | CCAAGGTGCACAAGGAATTT | detection of *tent* | |
|  | P1715 | CAATGTTTAATGCGGGTCCT |  |  |
|  | P2146 | AAGATGATGTAGCAGTAAGTATGGA | detection of *gyrA* | |
|  | P2147 | CTCTGAAGCCAATGTCCTTTT |  |  |
|  | P2142 | TTGAAGAATGTAAAGAGAGAGATGCTAC | detection of *rpoB* | |
|  | P2143 | GGGAAGTCACCCATAAAGACA |  |  |

pMRP1424 and pMRP1423 were built in the vector pAT19 and pMRP1421 into pMRP306, a derivative of pAT19 containing the constitutive promoter of the iota toxin gene, the cloning sites *Nco*I-*Pst*I, and the 3' part of the iota toxin gene ^1^

P1736-P2394 1500 bp sequencing of the additional insert in tent
